# Terahertz structured light: nonparaxial Airy imaging using silicon diffractive optics

**DOI:** 10.1038/s41377-022-01007-z

**Published:** 2022-11-17

**Authors:** Rusnė Ivaškevičiūtė-Povilauskienė, Paulius Kizevičius, Ernestas Nacius, Domas Jokubauskis, Kęstutis Ikamas, Alvydas Lisauskas, Natalia Alexeeva, Ieva Matulaitienė, Vytautas Jukna, Sergej Orlov, Linas Minkevičius, Gintaras Valušis

**Affiliations:** 1 Department of Optoelectronics, Center for Physical Sciences and Technology, Saulėtekio av. 3, Vilnius, 10257 Lithuania; 2 Department of Fundamental Research, Center for Physical Sciences and Technology, Saulėtekio av. 3, Vilnius, 10257 Lithuania; 3 Institute of Applied Electrodynamics & Telecommunications, Vilnius University, Saulėtekio av. 3, Vilnius, 10257 Lithuania; 4 CENTERA Labs., Institute of High Pressure Physics PAS, ul. Sokolowska 29/37, Warsaw, 01-142 Poland; 5 Department of Organic Chemistry, Center for Physical Sciences and Technology, Saulėtekio av. 3, Vilnius, 10257 Lithuania; 6 Institute of Photonics and Nanotechnology, Department of Physics, Vilnius University, Saulėtekio av. 3, Vilnius, 10257 Lithuania

**Keywords:** Terahertz optics, Imaging and sensing, Silicon photonics

## Abstract

Structured light – electromagnetic waves with a strong spatial inhomogeneity of amplitude, phase, and polarization – has occupied far-reaching positions in both optical research and applications. Terahertz (THz) waves, due to recent innovations in photonics and nanotechnology, became so robust that it was not only implemented in a wide variety of applications such as communications, spectroscopic analysis, and non-destructive imaging, but also served as a low-cost and easily implementable experimental platform for novel concept illustration. In this work, we show that structured nonparaxial THz light in the form of Airy, Bessel, and Gaussian beams can be generated in a compact way using exclusively silicon diffractive optics prepared by femtosecond laser ablation technology. The accelerating nature of the generated structured light is demonstrated via THz imaging of objects partially obscured by an opaque beam block. Unlike conventional paraxial approaches, when a combination of a lens and a cubic phase (or amplitude) mask creates a nondiffracting Airy beam, we demonstrate simultaneous lensless nonparaxial THz Airy beam generation and its application in imaging system. Images of single objects, imaging with a controllable placed obstacle, and imaging of stacked graphene layers are presented, revealing hence potential of the approach to inspect quality of 2D materials. Structured nonparaxial THz illumination is investigated both theoretically and experimentally with appropriate extensive benchmarks. The structured THz illumination consistently outperforms the conventional one in resolution and contrast, thus opening new frontiers of structured light applications in imaging and inverse scattering problems, as it enables sophisticated estimates of optical properties of the investigated structures.

## Abstract (web summary)

Demonstration of generation of terahertz structured light in a form of configurable Airy beam for imaging aims and 2D materials inspection using exclusively flat silicon diffractive optics.

## Graphical abstract

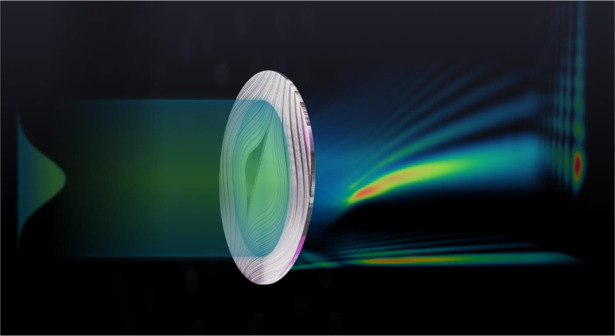

## Introduction

Structured electromagnetic fields have been shown to be viable in a variety of applications such as communication, metrology, and light-matter interactions^1,2^. Most advances are due to the rapid development of so-called nondiffracting electromagnetic beams^3^ which nowadays are applicable for a broad selection of wavelengths extending from visible^4^ to terahertz (THz) ranges^5^. Such popularity is caused by intriguing properties like diffraction and dispersion resistance^6^, self-healing^7,8^, self-acceleration^9–11^, etc. The applicability of nondiffracting beams can be significantly increased when their vortical^12,13^ and polarization properties^14^ are introduced^15^.

The family of nondiffracting beams has numerous relatives to the well-known Bessel beam. Elliptic Mathieu^16^, parabolic Weber^17^, Pearcey beams^18^ and self-accelerating Airy beams^9,19^ are less recognized, however, they can be found as more promising members in the family of nondiffracting illumination. The nondiffracting Airy beam manifests itself as a beam of a distinct parabolic propagation trajectory in the longitudinal plane^20^. The beam trajectory (or caustics) of nondiffracting electromagnetic fields can be shaped to any other form^21–23^.

These nonconventional states of light also can find their use also in imaging improvements^24–26^, laser microfabrication^27,28^, photonic communications^29,30^, tomographic^31^, light sheet^32^, and sub-THz microscopy^33^. High resolution is one of the main objectives of the imaging theory, and it is usually achieved with Gaussian illumination in high numerical aperture systems. However, a high numerical aperture automatically leads to bulky imaging systems. The lenses and conical prisms become rather massive, and various aberrations can be induced.

The solution to this problem is flat optics^34–37^, with Fresnel lenses^34,38^ and axicons^4,26^ being the most well-known examples. Flat optics elements can also enable efficient generation of Airy beams in both transmission and reflection geometries^39^ in a wide range of wavelengths, from optical^19,40^ to THz^41^. Flat optics reduces the thickness and weight of optical elements by exploiting diffraction. A spatial arrangement of sub or near wavelength thickness elements that locally phase-shifts a passing optical ray which leads to the constructive interference of the transmitted waves at the focal point. An implementation of this concept involves binary or multilevel diffractive elements where the phase delay is (*n* – 1)*t* for the material of refractive index *n* and local thickness *t* of the substructure^34^. Yet another implementation is based on the concept of metasurfaces using propagation or geometrical phase^37^ For example, diffractive gratings were realized by local changes in the geometrical phase of the structure comprised of individual metaatoms^42,43^. We note that both approaches to flat optics can be equally effective; see ref. ^34^.

THz waves stand out among other sources and frequencies, as they not only serve as a flexible platform for scalable photonic experimentation, but also provide a strong background for a wide variety of applications in communications, spectroscopy, and imaging systems for nondestructive inspection in security, medicine, and materials research^44,45^. Recent progress in the development of compact and robust room temperature THz sources^46^ stimulated strong demand for relevant flat optic advances with the aim of reducing the size of imaging or spectroscopic systems^37,42,43,47–49^.

Its direct implementation requires convenience under real operational conditions, optimization, and miniaturization of THz imaging systems with reduced power consumption. Since silicon can be assumed to be one of the most promising materials for the development of compact THz systems containing solid-state-based emitters, room temperature detectors and their arrays, an important role must be attributed to the development of compact flat optics, in particular, considering their further integration into imaging setups^44,50^. Moreover, because of the relatively long THz wavelength, a large variety of high-quality compact metasurfaces, amplitude, and phase elements can be flexibly manufactured in a wide cost range. These circumstances display THz range as a flexible platform and promising toy model for fundamental research in scalable flat optics readily applicable for other wavelengths of electromagnetic radiation.

In this work, for the first time, we demonstrate a compact and nonparaxial solution based exclusively on flat silicon diffractive optics for structured THz light generation in the form of a configurable Airy beam. Second, we expose its accelerating nature via THz imaging of partially obscured objects using an opaque beam block. Third, in contrast to conventional approaches, when under paraxial conditions a combination of a lens and a cubic phase (or amplitude) mask creates a nondiffracting Airy beam, we also demonstrate simultaneous lensless nonparaxial Airy beam generation in the THz range. Fourth, the designed Airy lens performance in the conventional generation of the Airy beam, when a phase element creates the spatial spectra of the nonparaxial Airy beam, and in the non-conventional beam generation when the nonparaxial propagation itself performs focusing of the phase mask, are revealed and investigated.

Fifth, it is displayed that the self-accelerating properties of both Airy beams allow for recording of a THz image of an object behind an obstacle; also, high quality of THz Airy images of a single object and images over a controllable placed obstacle is exposed. Sixth, the ability to inspect the quality of 2D materials such as stacked graphene layers is affirmed and its correlation with Raman spectroscopy data is established.

Seventh, the distinct role of structured THz illumination on the quality of nonparaxial THz Airy imaging is illustratively benchmarked in mode profile measurements, numerical simulations, and their comparison with Bessel and Gaussian beams. Eighth, the THz imaging setup contains only flat optical elements manufactured from a high-resistivity silicon substrate ablated by femtosecond laser pulses^51^.

## Results

### Design and fabrication of the nonparaxial flat optics elements

Our aim is to design a flat photonic element for imaging objects with THz illumination. Due to the rather long-wavelength *λ* and characteristic dimensions of imaged objects, the element must be a nonparaxial object. Thus, its performance is described by the Rayleigh-Sommerfeld diffraction integral^52^.

For our purpose, we devised a cubic phase profile

1 $$\varPhi _{\rm{AI}}({{{\boldsymbol{r}}}}) = a\left( {x^3 - y^3} \right)$$

(*a* = *π* × 10^7^ m^−3^) which we encoded in a flat element, where structural changes in the height of the elements contribute to phase changes less than 2*π*. The complex transmission function *T*(**r**) of a multi-level phase mask is defined as

2 $$T({{{\boldsymbol{r}}}}) = {{{\mathrm{exp}}}}\left( {i\frac{{2\pi }}{N}\Bigg\lfloor\frac{{N\varPhi \left( {x,y} \right)}}{{2\pi }} - {N}\Big\lfloor \frac{{\varPhi \left( {x,y} \right)}}{{2\pi }}}\Big\rfloor\Bigg\rfloor \right)$$

where *Φ*(*x*, *y*) = *Φ*_AI_ (**r**) and the brackets ⌊⌋ represent a round-down operation and *N* is the integer number of levels in the phase mask.

**Fig. 1:**
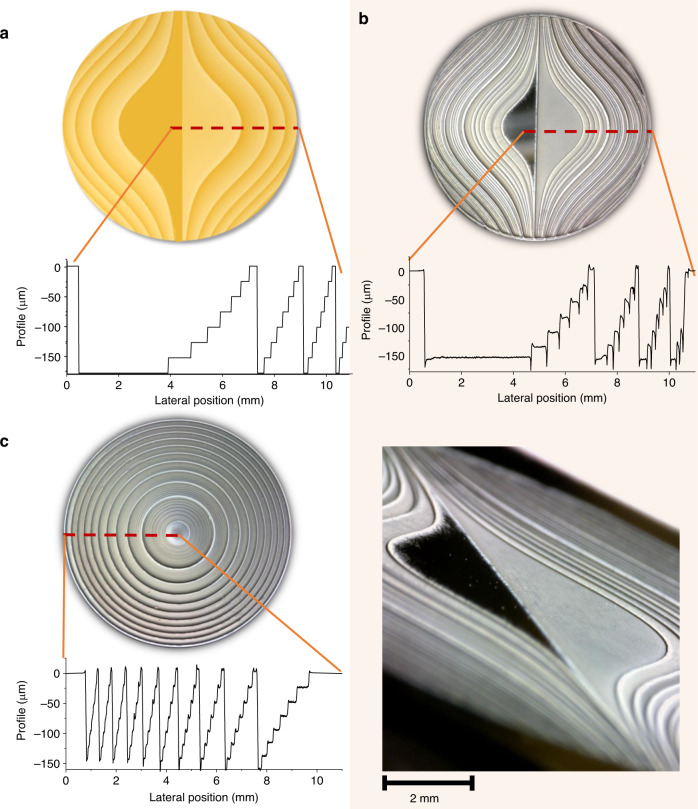
Designs of photonic elements under study. Spatial distribution of **a** designed nonparaxial Airy mask and its lateral cross-section; **b** fabricated silicon nonparaxial Airy element and its cross-section with the enlarged surface structure (bottom panel); **c** fabricated nonparaxial silicon zone plate with quadratic phase profile and its cross-section

The spatial profile of the designed nonparaxial Airy phase mask with *a* = *π* × 10^7^ m^−3^ and *N* = 8 is shown in Fig. 1a. This design represents a phase mask plate of eight levels (*N* = 8) of diameter 20 mm, which together with a zone plate (*f* = 1 cm) is dedicated to generate an Airy beam in the range of up to 10 mm.

To provide focusing abilities and additional management facilities for the engineered beam in the setup, we design and fabricate conventional zone plate with the phase of the transmission function being a quadratic phase function

3 $$\varPhi _{\rm{ZP}}({{{\mathbf{r}}}}) = \frac{k}{{2f}}\left( {x^2 + y^2} \right)$$

where *f* = 1 cm is the expected paraxial focus length. Similar designs and their performance were discussed in refs. ^51,53,54^.

Additionally, to generate Bessel THz beam a Fresnel axicon was designed with a linear phase function

4 $$\varPhi _{\rm{B}}({{{\mathbf{r}}}}) = \frac{{2\pi {{{\mathrm{sin}}}}\beta }}{\lambda }\sqrt {x^2 + y^2}$$

where *β* = 0.4 rad. In the end, we fabricated phase plates with linear, quadratic, and cubic phase profiles.

Fabrication was carried out on a high-resistivity silicon wafer with a refractive index of *n* = 3.46 and the wavelength of the electromagnetic radiation is *λ* = 0.5 mm corresponding to the frequency of 0.6 THz using the ultra-short pulsed laser ablation process^53,55^. Details can be found in the Materials and Methods Section.

The photographed spatial profile of the manufactured Airy lens, its cross section and the enlarged tilted photo of the Airy lens to visualize the levels, structural depth and quality of the surface of the fabricated element are depicted in Fig. 1b. For comparison, a picture of the 8-level laser ablated nonparaxial zone plate with a cross-section of the element as the inset is shown; see Fig. 1c.

One can note that no transverse distortions between the designed and fabricated elements are visible. However, only 7 phase levels can be resolved in the actual Airy element. For this reason, a phase difference of 0.6 rad (equal to 19.3 μm of height difference) between the designed and the fabricated elements was estimated. We have theoretically evaluated the influence of this phase mismatch and found it to be rather moderate, since it mostly causes a drop in the maximal intensity of about 40 percent, but it has almost no influence on the specifics of the spatial intensity distribution. Other elements were produced as intended.

As the devised elements are nonparaxial their performance is revealed using the Rayleigh-Sommerfeld integral with a spherical-point-source-based propagator. Details are given in the Materials and Methods Section. To illustrate the versatility of the operation of the designed flat optics for the 0.6 THz frequency, we studied the performance of the actual element with and without a nonparaxial zone plate.

**Fig. 2:**
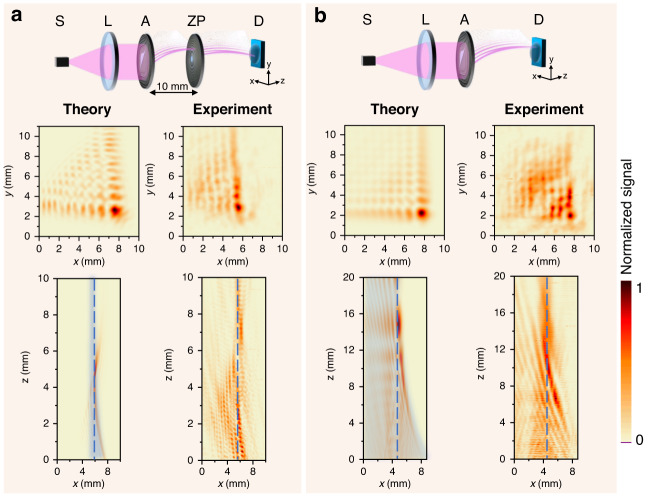
Numerically estimated and experimentally obtained intensity distributions of the nonparaxial Airy beam in the transverse (xy) (at the focal point) and in the longitudinal (xz) planes at 0.6 THz. S denotes a THz source, L is a lens, A stands for the Airy phase mask, ZP is a nonparaxial zone plate, D labels THz detector. Panel **a** – using Airy phase mask with an additional zone plate. The distance between the Airy lens and the ZP is 10 mm, and for the transverse plane scan the detector is placed in 7 mm from the ZP. The starting scanning point along the *z*-axis is 1 mm away from the ZP; panel **b** – using the Airy lens only. The starting scanning point along the *z*-axis is 1 mm away from the Airy lens. Distributions are normalized to the maximum value of the signal. A dashed blue line in bottom panels – guide for an eye to accentuate Airy beam which exhibits parabolic trajectory. A light blue shaded area in theoretical plot enlightens the reciprocal trajectories. Note that in case of nonparaxial Airy generation with mask without zone plate (panel **b**), the Airy profile is not distorted as in the case with zone plate (panel **a**) although the beam increases its dimensions as it propagates along the optical axis proportionally to *z*^2^. Reciprocal caustic trajectories following *x*∼1/*z* law are also nicely pronounced both in theory and experiment. As it is seen the experiments fit well the modelling results (panel **b**)

Simulations were performed using an incident Gaussian beam of radius *w*_0_ = 10 mm at the intensity level of 1/*e*^2^. The beam was collimated by the lens *L* and directed onto the Airy phase mask to generate structured THz light, see Fig. 2. In Fig. 2a the distance between the Airy mask and the ZP was *f* = 1 cm. The simulation area is 8.5 mm × 20 mm in the longitudinal (*xz*) plane.

First, we calculate the electromagnetic fields generated using the Airy phase mask and a zone plate; see Fig. 2a. The design of the zone plate was based on the paraxial assesment of the focal spot at *z* = 1 cm. The actual nonparaxial focal point was formed at the distance *z* = 0.85 cm.

The analysis of the beam propagation revealed that the beam behaves as expected; the trajectory of the generated Airy beam is parabolic, although some deviations from the paraxial trajectory are observed. Due to the nonparaxiality (we remind that the propagator is spherical-point-source-based), the transverse profile is slightly distorted on the edges. This is a distinct effect caused by the highly nonparaxial zone plate – strictly speaking, the optical Fourier transform is valid only in the paraxial regime, consequently, the nonparaxial beam profile is not a product of two independent profiles as it is in the paraxial case, see Methods. It should be noted that the parabolically bending nonparaxial Airy beam focuses approximately 5% further as expected at *z* = 1.05 cm.

Subsequently, we looked into the performance of the nonparaxial Airy generating mask without a zone plate. Due to the choice of the parameter *b*, see Eq. (5), the reciprocal trajectories, following the law *x* ∼ 1/*z* can be revealed in the theoretical plot within the acceptable range of coordinates and are clearly observed experimentally, see Fig. 2b. Interestingly, the Airy profile is less distorted than in the previous case, compared with Fig. 2a, though the beam increases its dimensions as it propagates along the reciprocal trajectory proportionally to *z*^2^, see Methods.

### Experimental verification of the nonparaxial designs

We start by benchmarking the fabricated THz phase element with experimental verification of its performance. Our aim here was to experimentally generate nonparaxial Airy beams with conventional parabolic and unconventional reciprocal trajectories. The principal optical schemes are shown in the top row of Fig. 2a, b.

The generation of the Airy beam was carried out experimentally using an electronic multiplier chain-based emitter (*Virginia Diodes*, *Inc*) to produce radiation of 0.6 THz frequency. Delivered through a converging lens (*L*, with *F* = 12 cm) it was collimated to illuminate the focusing elements arranged in different orders. The first setup is based on the Airy lens and zone plate combination and is shown in Fig. 2a, while the second setup uses only the cubic mask (Fig. 2b). The beam profiles along and perpendicular to the beam propagation direction were raster-scanned at a speed of 10 mm s^−1^. Recording of a 100 mm^2^ in *xy* plane and 160 mm^2^ in *xz* plane-sized images of the focused Airy and Gaussian beam with a pixel size of 0.1 mm^2^ at a scanning speed of 10 mm s^−1^ leads to a signal-to-noise ratio (*SNR*) of 570 for a setup with the single Airy lens and *SNR* = 1180 for the set-up containing the Airy lens and the zone plate.

Measurement was carried out in the area which was consistent with the simulation area. Typical experimental Airy beam profiles after the zone plate both in the transverse and longitudinal planes are presented in Fig. 2a. In general, the transverse intensity profile is skewed in the same way as expected due to the action of the nonparaxial focusing zone plate. The peak position in the transverse plane *xy* has a similar intensity distribution and is slightly shifted in the *y*-direction. In the transverse plane, we observe a parabolic trajectory, with small deviations from the design due to the fabrication. The main lobe of the generated Airy beam structure spans from 0 to 10 mm. Therefore, we can infer a good performance of the element.

Next, we remove the zone plate ZP and repeat the experiment. As one can see from Fig. 2b, the transverse distribution is now undistorted, with both arms of the structured THz radiation being perpendicular. Some minor deviations are observed, but they can be explained by the experimental implementation. The longitudinal profile shows the diffractive spreading of the Airy profile. Although it almost keeps its shape over a distance from 6 mm to 16 mm, the diameter of the beam increases proportionally to the square of the distance *z* from the element. It is worth noting that the Airy beam exhibits reciprocal self-bending during propagation in free space. Most importantly, we confirmed that the Airy phase mask can be used experimentally to create nonparaxial structured THz light with and without a zone plate. This becomes possible due to the nonparaxiality of the devised elements.

### Imaging of objects with nonparaxial structured illumination

For practical applications, it is important to benchmark the performance of the fabricated cubic nonparaxial phase mask in THz imaging applications using structured THz light illumination. Most notably, the nonparaxiality of the element allowed us to generate two versions of the Airy beams with different setups discussed above.

The first benchmark is a raster scan imaging of the nontransparent sample when the sample is illuminated by the nonparaxial Airy beam. As structured light illumination displays a self-accelerating bending structure, we studied its performance in THz imaging of the object behind an obstacle. The second case is related to THz transparent samples, where the observation of the object and the registration of its internal structure are a challenge due to low absorption, while the main attention needs to be focused on a phase change.

**Fig. 3:**
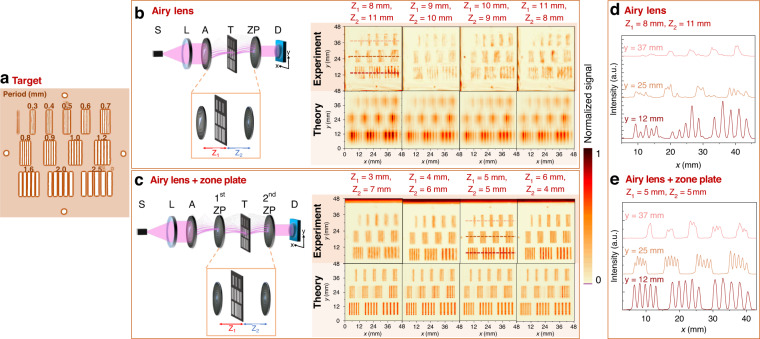
Imaging of non-transparent sample using nonparaxial Airy beam. Image of the target with different period slits (**a**). Performance of the nonparaxial Airy phase mask in THz imaging in two setups: with a single nonparaxial zone plate (**b**) and two nonparaxial zone plates (**c**). T indicates the imaged target. The color bar is normalized to the maximal value of the signal. *Z*_1_ and *Z*_2_ indicate distances between optical elements and the object. The first row on the right and the second row on the right present comparison of the experimentally obtained and numerically calculated object pictures for both setups, respectively. Cross-sections of the imaged objects (at positions denoted by dashed lines) are depicted in graphs (**d**) for setup with an Airy lens and a single nonparaxial zone plate and (**e**) for an Airy lens and two nonparaxial zone plates. Cross-sections of the recorded image (**d**) proving the 3.2 *λ* spacial resolution with more distorted target bars image; Cross-sections of the recorded image (**e**) with precise spatial frequency resolution reaching 1.6 *λ* with the contrast of 2 (a.u.)

For benchmarks of structured illumination imaging of nontransparent targets, we employed a structure, which we routinely use in THz imaging experiments^26^. It contains a number (5) of bars of different widths arranged in groups of the same number of bars. The target has three rows of bars; the first row is 5 groups of the 5 smallest bars, the middle row contains 4 groups of larger bars, and the last row has 3 groups of the largest bars. Period and size are marked in Fig. 3a.

The fabricated photonic element enables us to generate two distinctly different nonparaxial versions of the structured THz illumination, so we investigated and benchmarked the performance of the imaging application in two set-ups. The first one contained the single Airy lens (Fig. 3a) while the second one consisted of the Airy lens in combination with the nonparaxial zone plate (Fig. 3b). In both experiments, the target was scanned in raster with a velocity of 20 mm s^−1^ and a pixel size of 0.2 mm. The distance between the target and adjacent optical elements varied during the scanning process. In Fig. 3b panel, the red letter *Z*_1_ marks the distance between the Airy lens and the target, while the blue letter *Z*_2_ indicates the distance between the target and the zone plate. Accordingly, in Fig. 3c panel, the red letter *Z*_1_ marks the distance between the first zone plate and the target, and the blue letter *Z*_2_ indicates the distance between the target and the second zone plate. Since the Airy beam is nondiffractive, experimentation revealed that a similar image was recorded at all positions of the target object.

In the first case, the bar structure with the period of 1.2 mm is still distinguishable in the recorded images. Even the better spatial resolution and quality are achieved in the second setup with the Airy mask and the zone plate, see Fig. 3c, where the structure with the 0.8 mm period is still visible, and the contrast is 2 (*a*.*u*.).

A detailed comparison of spatial resolution and contrast is given in Fig. 3d, e, where the cross sections for each target bar (in places marked with red lines) are presented. It should be noted that a combination of the Airy mask with the zone plate provides spatial resolution reaching 1.6*λ*, while a simpler approach without the zone plate reaches only 3.2*λ* spatial resolution with the recorded images being more distorted.

We numerically evaluated the expected performance of the structured illumination under the same conditions as in the experiment. The results are given in Fig. 3. In general, the conventionally generated Airy beam was performed in a manner similar to that observed during the experiment. The resolution and contrast estimates agree well with the experimental observations.

However, we observed some discrepancies between the numerical estimate and the experiment when the nonparaxial Airy beam was generated non-conventionally. The experimental results demonstrate better contrast and resolution than the numerical estimation. Although the number of bars can be precisely estimated, the shape of bars looks slightly distorted in the numerics. It can be caused by the fact that the Airy beam with reciprocal trajectory spreads more in the numerical estimates than in the experiment and displays higher than experimental intensity. The reciprocal propagation and its direction were also the main cause of why in the virtual numerical experiment the shape of the bar is distorted, with one side of the bar looking more intense than the another one. The experimental realization can have some alignment imperfections; therefore, this effect is less pronounced in the recorded pictures.

Yet another intriguing feature of the self-accelerating structured THz illumination is the bending propagation trajectory. Results of the first imaging benchmarks induced an idea of a specific imaging experiment when an opaque obstacle is placed between the target and illumination.

In this set of experiments, we additionally employed a multilevel laser-ablated Fresnel axicon, which was previously benchmarked in THz imaging with the Bessel beam and allowed us to achieve a superresolution^26^. Given the nondiffractive nature of all contestants, this makes us wonder how those three set-ups can be compared under these rather unusual conditions.

Although the bending trajectory of the structured Airy illumination makes it easy to understand how the imaging behind the obstacle is possible, some additional comments are required in the case of nondiffracting Bessel beams. They can be represented as plane waves, whose wave vectors are located in the cone with angle *α*_*B*_^12,26^. For this reason, as we cover the target with an obstacle, we cover only a part of the plane wave components lying on the cone. The uncovered part of the spatial spectrum propagates at the cone angle *α*_*B*_ to the *z*-axis and reaches the region behind the obstacle. For this reason, we expect that part of the structured Bessel-type illumination is still able to reach the target. This property is observed many times in the literature under the moniker of the self-healing and self-recovery properties of the Bessel beam^8^.

**Fig. 4:**
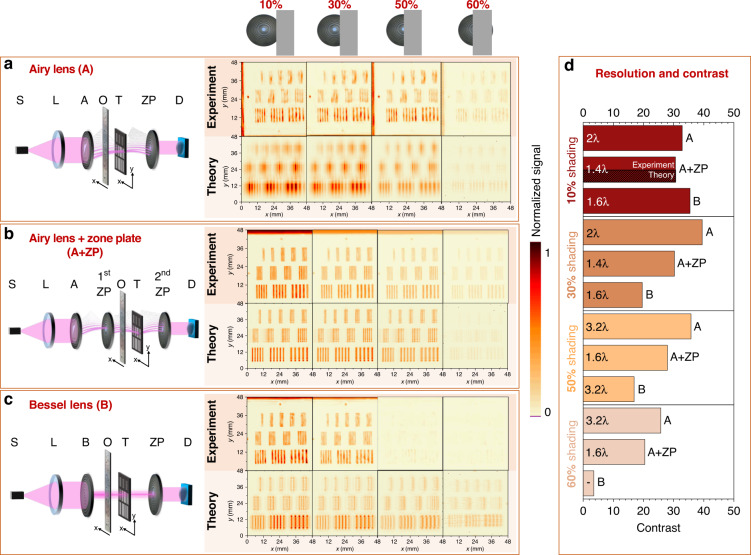
Benchmarking of structured nonparaxial illumination behind the obstacle. Performance of the nonparaxial Airy phase mask in THz imaging behind the obstacle in two setups: with the single nonparaxial zone plate (**a**) and two nonparaxial zone plates (**b**), where O denotes an obstacle. Performance of the nonparaxial Bessel phase mask in the same THz imaging behind the obstacle setup (**c**). The color bar is normalized to the maximum value of the signal for each setup. The position of the obstacle is depicted on top of four columns on the right, starting from the almost uncovered object (middle, ∼10% is covered) and ending with the most covered object (right, ∼60% is covered). Results of resolution and contrast benchmarks for different coverage of the structured illumination percentage (**d**). Resolution values are marked inside the colored bars, the contrast is given on the x-axis. For the sake of brevity, the numerical estimate is given only for one particular case, since in all other cases the results between theory and experiments are well-coincident within 2 percent accuracy. The contrast is defined as the ratio of the maximal and minimal intensity values

The experimental results of a sample imaging behind the obstacle are presented in Fig. 4. The obstacle was placed at the distance *z* = 1 mm behind the last element in the scheme before the object and other distances were selected optimally from the previous experimentation. A metal plate impermeable to THz radiation was used as an obstacle in three different setups. During the experimentation, an ever-increasing area of the optical element in front of the sample was shielded by this metal plate. The experimentation with structured THz illumination was repeated three times using different lens configurations in front of an obstacle: Airy lens only (Fig. 4a), the Airy lens in combination with the nonparaxial zone plate (Fig. 4b), and the Bessel lens (Fig. 4c).

As experimentation has revealed, see Fig. 4a, when a single Airy lens is employed, the image of the target is clear even when the optical element is almost completely covered by the metal obstacle. The smallest period of stripes, which is clearly resolved, is 1.2 mm. Our experimentation is in line with the numerical estimate presented in the same Figure. Some slight discrepancies might be caused by slight misadjustments in the system and slight imperfections in the position estimates. These results confirm the self-healing properties of the Airy beam and promise an opportunity to perform THz structured light imaging even when an opaque object is present.

The results are even more promising; see Fig. 4b when a combination of the nonparaxial cubic phase mask and the nonparaxial zone plate is employed. The slits of the target are still clearly visible, even if more than half of the illuminating element is covered. In this case, a surprisingly good resolution is still observed, indicating that the smallest period of stripes, which is clearly resolved, is 0.7 mm.

Lastly, we are curious how the Fresnel axicon will perform, as the structured THz illumination behind it is still nondiffracting and thus also self-recovering. During the experimentation with the Bessel lens (Fig. 4c), when small parts of the generating element are covered, the image of the target is still clearly recorded. In this case, the smallest period of stripes, which is clearly resolved, is 0.8 mm (1.6*λ*). Nevertheless, when a larger area of the element is covered, the image of the target is barely seen until only the noise is recorded. Numerical estimates demonstrate a similar behavior, although some images can be recognized in the background. This deviation is caused by some experimental uncertainties in the generated structured light illumination. Thus, the fabricated cubic phase plate shows better results in this benchmark than the Bessel generating element.

A summary of the performance benchmarks is given in Fig. 4d. First, we note that the performance of the single Airy phase mask decreases as we cover a larger part of the structured illumination, the spatial resolution changes up from 2*λ* to 3.2*λ* while the contrast fluctuates in the range of 30 (a.u.). Surprisingly, in the second experimentation, we did observe the even better performance of the Airy phase mask in conjunction with the zone plate - the spatial resolution does not depend on the covered percentage. It is 1.6*λ*, and thus it is better than in the first experiment. The contrast was slightly less than for the case without the zone plate and did drop as we cover the illuminating element from 30 to 20. Lastly, the spatial resolution of the illumination of the Bessel beam decreased from 1.6*λ* to 3.2*λ*. Most noticeably, the contrast did drop drastically until no images were recorded for the largely covered illumination. An element covered by 30% results in a strongly reduced contrast for the case of the Bessel illumination, while the cases of the Airy and the Airy with the zone plate demonstrate the contrast less dependent on the block percentage.

Numerical estimates agree well with the experimental observations; for the sake of brevity, we present them only for one particular setup, where the agreement is the largest; see Fig. 4d. This is the second setup, see Fig. 4b, numerically obtained contrast does not deviate more than 5% from the experimental one.

Deviations are larger in the first set-up, though the dependence on the percentage of the covered part is similar. In the third case, see Fig. 4c, numerical contrast is smaller when 10% and 30% of the element are blocked, and it becomes equal to that in the experiment for the latter case indicating thus good agreement between the experiments and simulations.

### Inspection of thin 2D samples with nonparaxial THz structured light illumination

These promising results of our first two benchmarks have motivated us to study the performance of structured nonparaxial Airy THz illumination in the imaging of thin 2D samples. For this experimentation, we chose graphene as a highly interesting material for THz radiation. Graphene is one of the most popular and widely used two-dimensional materials consisting of a single layer of carbon atoms arranged in a two-dimensional honeycomb lattice nanostructure of only 0.3 nm thickness. Graphene is distinguished from other materials due to its specific properties: good electrical and thermal conductivity etc. In this benchmark, we have investigated in total 5 different graphene samples, each containing 1, 2, 3, 4 and 5 graphene layers placed on a high resistivity silicon substrate. Bare Si was used as a reference sample.

**Fig. 5:**
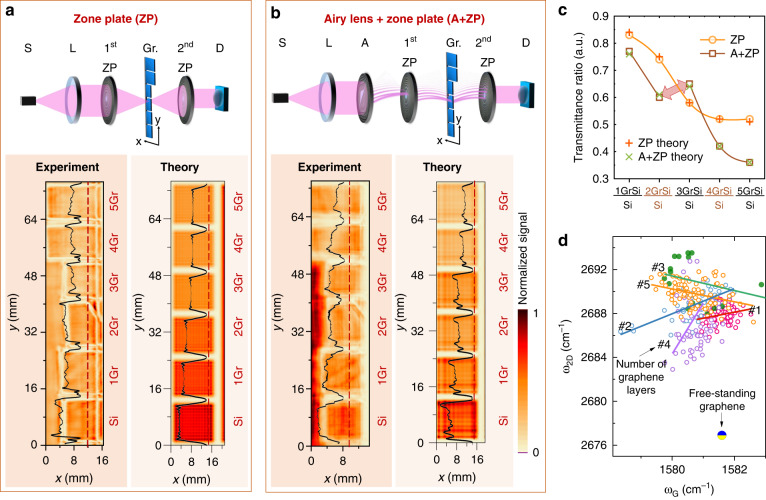
Imaging using nonparaxial THz structured light illumination. The sample under test consists of different numbers of graphene layers denoted on the graph. The THz illumination is produced using different configurations of lenses: (**a**) two zone plates and (**b**) an Airy phase mask combined with two-zone plates. Setups are depicted at the top of segments (**a**) and (**b**). Gr denotes the imaged sample with graphene layers. Experimentally obtained and numerically calculated imaging results are presented below. The color bars are normalized to the maximum signal. The dashed red line indicates the position of cross-sections of the recorded and calculated intensities given in the left bottom corner. Transmittance ratio of all samples for two different configurations of lenses (**c**). Correlation between Raman 2D and G peak positions for 1–5 graphene layers (**d**). Blue/yellow point marks free-standing graphene

All samples were investigated using the same setup, which we discussed earlier, with two different configurations of lenses: two-zone plates (Fig. 5a) and the Airy lens in combination with zone plates (Fig. 5b).

The results of the experiment and the numerical modeling for different numbers of graphene layers are given on the right side of the (a) and (b) parts of Fig. 5. The first setup does not generate the structured THz illumination, whereas the second one delivers the self-accelerating nonparaxial Airy beam.

In the first setup, under Gaussian illumination, the intensity decreases with an increase in the number of graphene layers. As expected, the drop is more or less linear for the first three graphene samples, while samples with 4 and 5 layers experience a weaker dependence; see Fig. 5c. Under structured THz Airy illumination, the situation becomes puzzling due to the pronounced nonmonotonic dependence: initially, the drop in transmission can be observed for the samples with 1 and 2 graphene layers, as expected; however, the unexpected increase in transmission is observed in the sample with 3 graphene layers. i. e., nonparaxial structured illumination is transmitted better through the sample than the conventional one. Regarding the sample with 4 graphene layers, the transmission drops well below that of the Gaussian illumination, and, more importantly, the sample with 5 graphene layers is now can clearly be distinguished from the sample with 4 layers which are in sharp contrast to the Gaussian case.

This issue can successfully be resolved taking into account the effects related to the light polarization, i. e., reasoning about the transmissivity of *s*- and *p* -polarized light (perpendicular and parallel) through a stratified medium (i. e., through a dielectric slab). First, the slab is a minuscule resonator with multiple reflections from the outer and inner surfaces that separate materials. Thus, any small deviation in the height of the sample or in the refractive indices of graphene or the Si wafer will have an imminent effect on the recorded transmissivity. For the paraxial illumination, there is no difference between the transmission of the incident polarization. However, the difference does appear for the nonparaxial illumination, as the transmission through the stratified medium behaves differently for plane wave components with large angles of incidence. Details are presented in the Supplementary Materials.

Therefore, one can assume that structured nonparaxial illumination not only ensures better spatial resolution but is also more sensitive to changes in the optical phase due to refractive indices and their variation in the stratified media. As the self-accelerating illumination has more spatial components with large transverse wave vectors, it distinguishes changes in optical parameters of the sample better than the conventional Gaussian illumination as given in Fig. 5a–c. Most importantly, a combination of these two signals together enables us to solve the inverse scattering problem^56^; i. e. we can make an educated and sophisticated guess to determine the height or effective dielectric constant of the sample.

To confirm the hypothesis, we have performed Raman spectroscopy of the graphene samples, of which two characteristic spectral bands, G and 2D, are plotted in Fig. 5d. It turns out that the samples contain distinct groups of graphene layers that behave differently. Due to graphene layer interaction with Si substrate or with other graphene layers, it can be mechanically strained. Second, graphene can experience electron depletion and other changes as a result of its deposition on the Si substrate. The existence of mechanical strain and doping can be exposed by performing the Raman mapping and examining the correlation analysis of the frequency position of the G and 2D bands; more details on Raman experiments can be found in the Supplementary Material. For the free-standing graphene sample, two characteristic bands, G and 2D, are located in the Raman spectra around 1580 cm^−1^ and 2690 cm^−1^, respectively. These values can vary depending on technological conditions in deposition processes or a number of the deposited layers as it is seen in distributions of *ω*_*G*_ and *ω*_2*D*_ of each sample depicted in Fig. 5d. Statistical dependency is accumulated from the single point data and a linear approximation as depicted on the plot. Zero-point indicates free-standing graphene and it is considered as reference (*ω*_*G*_ = 1581.6 ± 0.2 cm^−1^; *ω*_2*D*_ = 2676.9 ± 0.7 cm^−1^). The closer this *ω*_2*D*_ /*ω*_G_ distribution is to the zero point, the less this layer is affected by mechanical stress. As it is seen, the samples are strained differently. As can be seen, the 2D and G peak frequency distribution of the sample with 3 graphene layers is the farthest from the zero point, and its slope is −0.66 ± 0.5, which can be associated with doping effects. Inclusion of these effects together with light polarization in the modeling allows them to fit well with the experimental data, as can be seen in Fig. 5c and enabling thus to explain the increase in transmittance in the structured THz light experiment; see the Supplementary Materials for details. Therefore, it can be assumed that THz structured light can be assumed as a convenient and contactless tool for examining the properties of 2D materials and can also be extended to other materials systems or low-absorbing objects.

A good benchmark for the estimation of structured THz light illumination is *SNR*. For the conventional Gaussian-like illumination, we found the *SNR* to be around 118, meanwhile when the cubic phase mask creates a nonparaxial Airy beam the *SNR* is recorded to be almost three times higher *SNR* = 326. This is yet another indication that the structured illumination enables us to achieve significantly better resolution in the inspection of graphene layers.

## Discussion

It was demonstrated that the THz light can be shaped into structured self-accelerating and nonlinearly propagating nonparaxial radiation using high-resistance silicon diffractive optics prepared by femtosecond laser ablation technology.

It was shown that the designed element due to its nonparaxiality can simultaneously generate two distinct types of nonparaxial structured THz light – nondiffracting nonparaxial Airy beam with a parabolic trajectory and an expanding Airy beam with a reciprocal trajectory – in the same optical setup. Generation of these distinct three-dimensional field patterns was experimentally confirmed at 0.6 THz frequency and applied in THz imaging experiments.

Employing a sensitive, 90 nm CMOS technology-based THz detector signal-to-noise ratio has reached a value of 570 for the setup with the single Airy lens and that of 1180 for the setup with the Airy lens and the zone plate combination. Experiments indicated that, compared to only the Airy lens, the combination of the Airy lens and a nonparaxial zone plate results in twice better spatial resolution – 0.8 mm (1.6*λ*) period bars are visible.

THz imaging behind the obstacle was explored both numerically and experimentally in different obstacle geometries. It was validated that the Airy lens is superior in comparison to other illuminating elements, even if the ∼60% of the optical element was covered by an obstacle, the image of the target was still clear and exhibited a resolution of 3.2*λ* and the contrast of around 26 (*a*.*u*.). Even better results were achieved for parabolically bending structured THz illumination: When the ∼60% of the optical element was covered, the resolution did not decrease and was twice (1.6*λ*) as good as for the reciprocally bending structured THz illumination (3.2*λ*) with slightly lower contrast to 21 (*a*.*u*.).

With an increase in the blocked part, the contrast of the image experiences almost no changes in settings that contain bending illumination, while for the Bessel lens the contrast drops drastically from 35 (*a*.*u*.) to 3 (*a*.*u*.). Thus, although both Airy and Bessel illumination is nondiffractive, the bending propagation of the structured THz light provides an opportunity to image samples behind an obstacle with sufficient resolution and reasonable contrast.

To summarize, we presented the results of compact silicon flat optics-based THz structured light generation and its comprehensive benchmarks of various structured nonparaxial THz illumination-Bessel-like and self-accelerating along different paths. Our results indicate not only the good performance of the devised flat optics but also herald their potential in the applications of structured THz light in imaging and inspection of the quality of 2D materials e.g., stacked graphene layers. As the Raman spectroscopy indicates optical characteristics of the samples are different due to the strains, doping, depletion, etc., the structured Airy THz illumination gives an additional possibility to evaluate optical properties of stratified samples when combined with the conventional approach. It is in sharp contrast to the Gaussian illumination, where these features are indistinguishable. Extensive benchmarking allows to infer that the structured light consistently outperforms the classical Gaussian beam in THz imaging in such metrics as resolution and contrast allowing thus its expansion of applicability in rather complex investigation scenarios.

## Materials and methods

### Theoretical background

A distinct feature of the nondiffracting non-apertured paraxial Airy beam is its cubic spectral phase distribution *S*(*k*_*x*_, *k*_*y*_)^9,32^

5 $$S(k_x,k_y) = {{{\mathrm{exp}}}}\left[ {\frac{i}{3}(2\pi b)^3\left( {k_x^3 + k_y^3} \right)} \right]$$

where *k*_*x*_, *k*_*y*_ are transverse components of spatial spectra, *x*, *y* denote spatial coordinates, and *b* is some characteristic acceleration value. A Fourier transform of the spatial spectrum results in the following expression for the electromagnetic field in the focus

6 $$\begin{array}{l}{\int}_{ - \infty }^{ + \infty } {{{{\mathrm{exp}}}}\left[ {\frac{i}{3}(2\pi b)^3\left( {k_x^3 + k_y^3} \right)} \right]{{{\mathrm{exp}}}}[ - 2\pi i(k_xx + k_yy)]{{{\mathrm{d}}}}k_x{{{\mathrm{d}}}}k_y} \\ = \frac{1}{{b^2}}{{{\mathrm{Ai}}}}\left( { - \frac{x}{b}} \right){{{\mathrm{Ai}}}}\left( { - \frac{y}{b}} \right)\end{array}$$

Expression in Eq. (6) is a solution of a paraxial diffraction equation.

The propagation-dependent paraxial expression is given by

7 $$\begin{array}{l}U(x,y,z)\quad \approx {{{\mathrm{exp}}}}\left[ { - 2\pi i\lambda z\left( {\frac{1}{{2\pi b}}} \right)^3\left( {x - \frac{{z^2}}{{4b^3k^2}}} \right)} \right]\\ \quad \quad \qquad \qquad \;\; \times\, {{{\mathrm{exp}}}}\left[ { - 2\pi i\lambda z\left( {\frac{1}{{2\pi b}}} \right)^3\left( {y - \frac{{z^2}}{{4b^3k^2}}} \right)} \right]\\ \quad \quad \qquad \qquad \;\; \times\, {{{\mathrm{Ai}}}}\left[ {\left( {\frac{x}{b} - \frac{{z^2}}{{4b^4k^2}}} \right)} \right]{{{\mathrm{Ai}}}}\left[ {\left( {\frac{y}{b} - \frac{{z^2}}{{4b^4k^2}}} \right)} \right]\end{array}$$

It reveals the main feature of the nondiffracting Airy beam: the accelerating parabolic trajectory 4*b*^3^ *k*^2^ *x* = *z*^2^ of the dominant intensity peak. Most notably, the Airy cross-section is preserved during propagation.

Without loss of generality, a finite energy expression of the Airy beam is obtained by the introduction of the Gaussian envelope to the spatial spectra in Eq. (5). In other words, the cubic phase mask for the spatial spectra is essential in the engineering of the Airy beam.

We note that the designed element can also shape the incident radiation into a diffracting “caustic” beam when used without a lens^22,57^. In the paraxial regime, it is easily demonstrated by using the stationary phase method to evaluate the Fresnel integral^57^

8 $$U(x,y,z) \cong {{{\mathrm{Ai}}}}\left[ {\frac{{\left( {k + 2(2\pi b)^3xz} \right)^2}}{{2^{4/3}z^2(2\pi b)^4}}} \right]{{{\mathrm{Ai}}}}\left[ {\frac{{\left( {k + 2(2\pi b)^3yz} \right)^2}}{{2^{4/3}z^2(2\pi b)^4}}} \right]$$

leading to the reciprocal trajectory of the Airy beam *z* = −*k*/(16*π*^3^ *b*^3^ *x*). There are two differences in comparison to the conventional realization, see Eq. (7): 1) the reciprocal trajectory of propagation and 2) the quadratic spreading with the propagation distance *z* due to the presence of the factor 1/*z*^2^ in Eq. (8). Here, we note that in the paraxial regime the trajectories of the caustics produced with and without the lens do not overlap, as the coefficient *b* enters equations for trajectories differently: 4*b*^3^ *k*^2^ *x* = *z*^2^ for the conventional case and for unconventional generation as *z* = −*k*/(16*π*^3^ *b*^3^ *x*). It is important to underline that the selected parameters ensure a simultaneous generation of two different types of Airy beam.

### Numerical methods

In general, the choice of parameter *b* in Eq. (5) usually describes paraxial masks. However, in our case, we choose $b = \root {3} \of {{3A}}/(2\pi ) = 50$ m^−1^ therefore, numerical verification of its performance and validation of the trajectories of Eqs. (7, 8) was performed. We start by recalling the Rayleigh-Sommerfeld diffraction integral^52^.

9 $$U({{{\mathbf{r}}}}_1) = \frac{1}{{i\lambda }}{\int}_{S_{{{\mathrm{A}}}}} {U_{{{{\mathrm{inc}}}}}({{{\mathbf{r}}}}_0)T({{{\mathbf{r}}}}_0)\frac{{{{{\mathrm{exp}}}}\left[ {{{{\mathrm{i}}}}k\left| {{{{\mathbf{r}}}}_{01}} \right|} \right]}}{{|{{{\mathbf{r}}}}_{01}|}}{{{\mathrm{cos}}}}\left( {r_{01},n} \right)dS}$$

where *U*(**r**_1_) is the field in the observation plane, *U*_inc_ (**r**_0_) is the incident field in the diffraction plane, *T*(**r**_0_) is the transmittance of the object. The coordinates of the observation plane are **r**_0_ = (*x*_0_, *y*_0_, *z* = 0) and the coordinates of the observation plane are **r**_1_ = (*x*_1_, *y*_1_, *z* = *z*_*o*_), the vector **r**_01_ is the distance between two points in these planes, and n is normal to the surface of the object. Integration is performed over the surface of the element *S*_A_. Based on this consideration we have employed a propagator using spherical point sources to numerically model propagation of the electromagnetic field within the system.

### Fabrication of silicon optics

The core material for production was 500 μm thick high-resistivity silicon wafer with a refractive index of *n* = 3.46. It was ablated employing a femtosecond pulse duration laser, which was a Pharos SP (Light Conversion Ltd.). It generated a maximum power of *P* = 6 W at *λ* = 1030 nm wavelength with a tunable repetition rate of 4–200 kHz and an output beam size of 9 mm at the intensity level exp(−2). For the optimal ablation process, the shortest pulse duration of *τ* = 156 fs was employed keeping *P* = 5 W average power at 50 kHz repetition rate making thus *E* = 100 μJ energy per pulse. The pulse overlap density was established at 100 pulses per millimeter, while the spot size was approximately 20 μm. The amount of material removed in one pass was about 0.86 μm deep. Although the material ablation rate was not high – it was 1.257 μm^3^ s^−1^ we used these optimal parameters to avoid excessive burning of the silicon substrate and to produce relatively smooth ablated surfaces with roughness less than 2 μm. In general, a recipe for technological quality of flat silicon optical elements production relies on a rational balance between the fabrication duration and optimal ablation rate aiming to minimize material roughness and avoid silicon oxidation.

### The detection scheme

In both benchmarks, the focused beam was registered with the raster scanning technique in the *xyz* directions using the Si-CMOS field effect transistor manufactured using 90 nm foundry technology and integrated with a 670 µm-diameter log-spiral THz antenna. An additional Si substrate lens with a diameter 12 mm and height 6.8 mm is used for efficient coupling between the antenna and the THz radiation, which is directed from the substrate side. Before the experiment, the THz sensor was investigated, and it was determined that it exhibits rather a flat responsivity over the 0.1–2 THz range with values of optical responsivity and noise equivalent power of around 40 mA W^−1^ and 42 pW Hz^−0.5^, respectively^58^.

## Supplementary information

Supplemental material

Transmittivity for p-polarization

Transmittivity for s-polarization

Ratio of the transmission
