# Supplementary material for: Terahertz structured light: nonparaxial Airy imaging using silicon diffractive optics
Source: Light Sci Appl. 2022 Nov 17;11:326. doi: 10.1038/s41377-022-01007-z (PMC9668966; doi:10.1038/s41377-022-01007-z)
Supplement: Supplementary file 1 — Supplemental material [file 41377_2022_1007_MOESM1_ESM.pdf]

Supplementary Information for Terahertz structured light: nonparaxial Airy imaging using silicon diffractive optics

Rusnė Ivaškevičiūtė-Povilauskienė[1*], Paulius Kizevičius[2], Ernestas Nacius[2], Domas Jokubauskis[1], Kęstutis Ikamas[3], Alvydas Lisauskas[3,4], Natalia Alexeeva[1], Ieva Matulaitienė[5], Vytautas Jukna[2], Sergej Orlov[2], Linas Minkevičius[1,6] and Gintaras Valušis[1,6]

[1*]Department of Optoelectronics, Center for Physical Sciences and Technology, Saulėtekio av. 3, Vilnius, 10257, Lithuania.
[2]Department of Fundamental Research, Center for Physical Sciences and Technology, Saulėtekio av. 3, Vilnius, 10257, Lithuania.
[3]Institute of Applied Electrodynamics & Telecommunications, Vilnius University, Saulėtekio av. 3, Vilnius, 10257, Lithuania.
[4]CENTERA Labs., Institute of High Pressure Physics PAS, ul. Sokolowska 29/37, Warsaw, 01-142, Poland.
[5]Department of Organic Chemistry, Center for Physical Sciences and Technology, Saulėtekio av. 3, Vilnius, 10257, Lithuania.
[6]Institute of Photonics and Nanotechnology, Department of Physics, Vilnius University, Saulėtekio av. 3, Vilnius, 10257, Lithuania.

*Corresponding author(s). E-mail(s): rusne.ivaskeviciute@ftmc.lt;

1 Theoretical background of analysis of Airy THz imaging in stratified medium samples

Puzzling behaviour of the sample with graphene layers did require some investigation. First of all, during the deposition on the Si wafer, the graphene layers

could be modified due to the interaction with the substrate. The presence of the substrate and the number of the accompanying layers have influence on the optical constant of the graphene [1–3]. Uniaxial or biaxial strain can also appear, which will lead to modulation of conductivity and refractive index [4]. However, the presence of graphene could modify the conductivity of the Si wafer; thus, its optical parameters could also be somewhat modified [5, 6]. Revealing possible reasons of the situation observed in the experiment is the main motivation of the text below.

Our considerations are based on the basics of propagation of electromagnetic waves through the stratified medium [7, 8]. The characteristic matrix $\boldsymbol{M}(z)$ is shown to be

$$\boldsymbol{M}(z) = \begin{bmatrix} \cos\beta & -\frac{\mathrm{i}}{p}\sin\beta \\ -\mathrm{i}p\sin\beta & \cos\beta \end{bmatrix}, \tag{1}$$

where k_0 is the wave vector in vacuum, $\beta = k_0 n z \cos\theta$, n is the complex refractive index of the medium, z is the distance between two surfaces of the dielectric film, and θ is the angle at which the plane wave propagates inside the film. For the TE (s-polarized) wave, the parameter p is

$$p = \sqrt{\frac{\varepsilon}{\mu}}\cos\theta. \tag{2}$$

It should be replaced by the parameter q if the incident polarization becomes TM (p-polarized)

$$q = \sqrt{\frac{\mu}{\varepsilon}}\cos\theta \tag{3}$$

The effective characteristic matrix of a three-phase system is given by

$$\boldsymbol{M}(z_2) = \boldsymbol{M}_1(z_1)\,\boldsymbol{M}_2(z_2 - z_1) = \begin{bmatrix} m'_{11} & m'_{12} \\ m'_{21} & m'_{22} \end{bmatrix}, \tag{4}$$

where phase 1 occupies space from $z = 0$ to $z = z_1$ and phase 2 extends from $z = 1$ to $z = z_2$. This procedure is repeated as many times as many strata are present in the sample.

The thickness of graphene layers is small; hence, for this situation, we get an approximation

$$\boldsymbol{M}_2 = \begin{bmatrix} 1 & -\frac{\mathrm{i}\beta_2}{p_2} \\ -\mathrm{i}p_2\beta_2, & 1 \end{bmatrix} = \begin{bmatrix} 1 & -\frac{\mathrm{i}}{p_2}k_0 n_2 \delta z_2 \cos\theta_2 \\ -\mathrm{i}p_2 k_0 n_2 \delta z_2 \cos\theta, & 1 \end{bmatrix}, \tag{5}$$

which can be generalized for N graphene layers as

$$\boldsymbol{M}_{2,N} = \begin{bmatrix} 1 & -\mathrm{i}k_0 \sum_{j=2}^{N} \frac{1}{p_j} n_j \delta z_j \cos\theta_j \\ -\mathrm{i}k_0 \sum_{j=2}^{N} p_j n_j \delta z_j \cos\theta, & 1 \end{bmatrix}, \tag{6}$$

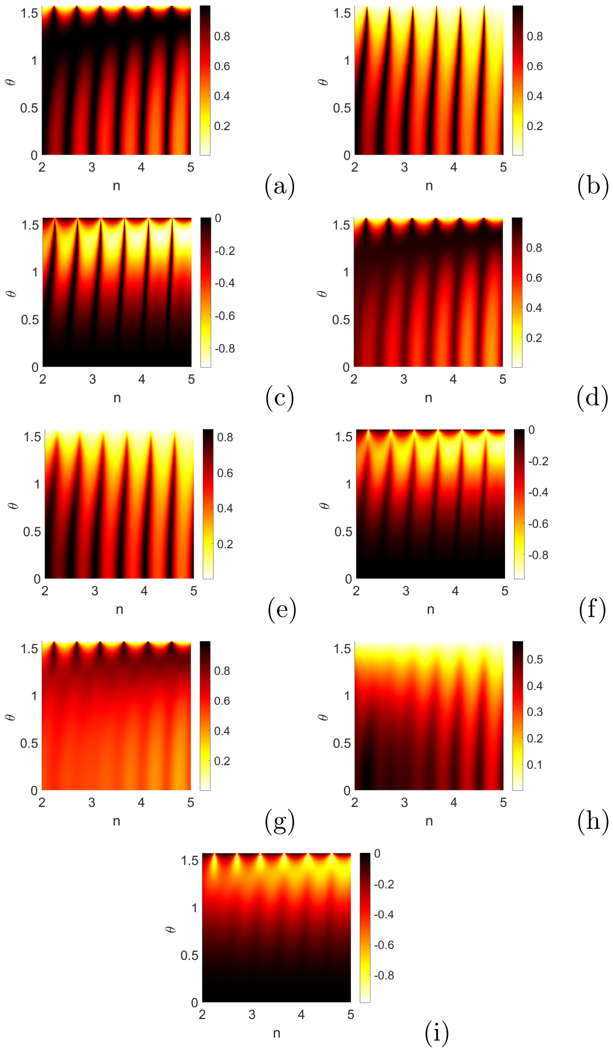

Fig. S1 Transmittivity of the stratified layer on the refractive index n of the substrate and angle of incidence θ for the (a,d,g) p-polarized and (b,e,h) s-polarized THz radiation of the frequency $\omega = 0.6$ THz, the thickness of the film is $d = 500$ µm. (c,f,i) Comparison of absolute numbers of the transmittivity between s and p polarizations. The graphene is (a-c) depleted and $n_{\mathrm{gr}} = 1.68$, (d-f) $n_{\mathrm{gr}} = 223 + 223i$, (g-i) $n_{\mathrm{gr}} = 500 + 500i$.

Assuming homogeneity of individual graphene layers simplifies the expression and the characteristic matrix is

$$\boldsymbol{M} = \begin{bmatrix} \cos\beta_1 - \frac{p_2\beta_2}{p_1}\sin\beta_1 & -\frac{\mathrm{i}\beta_2}{p_2}\cos\beta_1 - \frac{\mathrm{i}}{p_1}\sin\beta_1 \\ -\mathrm{i}p_1\sin\beta_1 - \mathrm{i}p_2\beta_2\cos\beta_1 & \cos\beta_1 - \frac{p_1\beta_2}{p_2}\sin\beta_1 \end{bmatrix}. \tag{7}$$

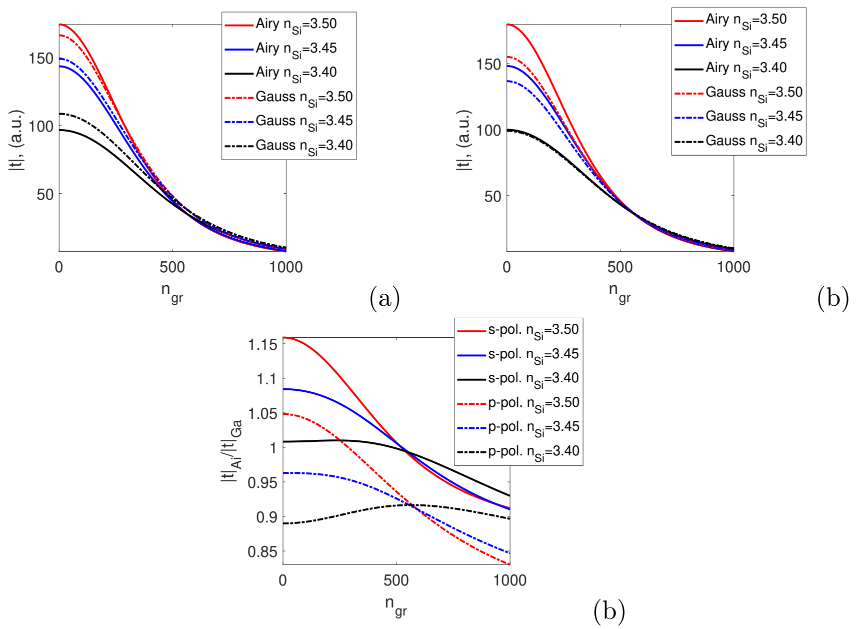

Fig. S2 Transmission of the nonparaxial Gauss (dotted line) and Airy (continuous line) illumination through the stratified layer with graphene layer of refractive index $n_{\rm gr}$ for the (a) s-polarized and (b) p-polarized THz radiation of the frequency $\omega = 0.6$ THz, the thickness of the Si wafer is $d = 500$ µm, refractive index is (black) $n = 3.4$, (blue) $n = 3.45$ and (red) $n = 3.5$. The thickness of a single graphene layer is $d_g = 0.3$ nm. (c) Ratio of the transmission of the nonparaxial Airy illumination normalized by the transmission of the nonparaxial Gauss illumination for the same case as previously, continuous line is p polarization, s polarization is dotted line.

The transmission coefficient of the stratified medium is given by

$$t = \frac{2p_1}{(m'_{11} + m'_{12}p_l)\, p_1 + (m'_{21} + m'_{22}p_l)}, \tag{8}$$

Once again, the expressions for the p polarization are obtained by replacing p with q. In particular, for thin 2D materials, the transmittivity depends on the thickness, number of layers, or absorption of the material only for very large values of the refractive index $n_{\rm gr}$.

The refractive index of the graphene changes in very wide range of values, the dielectric constant of the N graphene layers is seen as

$$\varepsilon_r(\omega) = 2.5 + i\frac{\sigma(\omega)}{\varepsilon_0 \omega d}, \tag{9}$$

where $\sigma(\omega)$ is the conductivity of the graphene, d is thickness and ε_0 is the permittivity of the vacuum. Some graphene samples can reach refractive indices up to $n_{\rm gr} = 1000 + 1000$i [1–3], on the contrary, the fully depleted graphene will reach values of only $n_{\rm gr} = 1.68$.

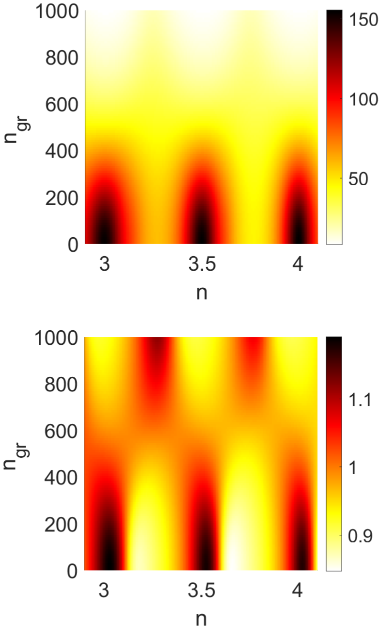

(a)

(b)

Fig. S3 (a) Transmission distribution T_s of the s polarized Gaussian beam through the stratified medium on the effective refractive index n of the Si substrate and on the normalized conductivity σ/d of the graphene. (b) Distribution of the ratio $T_s^{\mathrm{Airy}}/T_s^{\mathrm{Gauss}}$ between transmissions of the s polarized Gaussian T_s^{Gauss} and Airy T_s^{Airy} beams. The frequency is $\omega = 0.6$ THz, the thickness of the film is $d = 500$ µm.

.

First, we investigate the effect of graphene layers on transmittivity t, which is maximized when the denominator is maximized. The thickness of the graphene layers is very small and, as it turns out, the number of layers does not greatly influence transmittivity. For unusually high values of the refractive index $n_{\mathrm{gr}} > 200$, a local maximum is observed that is angle dependent, and the transmittivity starts to depend on the number of layers N. These values are common in the THz regime for metal-like substances. Because of the very thin layers, absorption does not greatly influence the transmission of the sample. Thus, the metallic behavior results in an angle-dependent transmission. As the literature reveals such behaviour can be caused by a variety of reasons.

Next, we investigate how the optical properties of the substrate (wafer) in combination with the optical properties of the graphene affect transmittivity, see Fig. S1. We fixed the refractive index of graphene n_{gr} to three distinct values: the value of a freestanding graphene (Fig. S1(a-c)), the semimetalic value (Fig. S1(e-f)) and the metallic value (Fig. S1(g-i)), and we changed the refractive index n of the substrate in the stratified sample. The real part of the refractive index (as well as the thickness of the sample) causes oscillatory changes in the transmittivity because the effective optical path is directly affected by these variables. Second, for the paraxial illumination, the angle of

the incidence (angle in phase 1) $\theta_1 \approx 0$, therefore, the angles θ_2 and θ_3 for remaining two phases via the Snell's law are also small; consequently, both light polarizations are transmitted equally, see (Fig. S1 (c,f,i)). However, a situation for the nonparaxial case becomes nontrivial, as depending on the film thicknesses and the refractive indices, difference in phases and angles can clearly be resolved. Thus, from the transmission values of the wafer, we can deduce its thickness and/or refractive index. Second, although the graphene layers are thin, the refractive index of the graphene has a distinct effect on the polarization-dependent angle-resolved transmittivity of the sample. As the graphene becomes metallic, the p polarization is transmitted better for larger values of incident angles θ, while the s polarization is transmitted better for smaller ones. This becomes even more pronounced, when the refractive index n_{gr} reaches values of $n_{\mathrm{gr}} = 1000 + 1000i$, see the Supplementary animations S1a and S1b.

Lastly, we numerically estimate the actual transmission of nonparaxial THz radiation. A numerical estimate was performed using an expression

$$U(\mathbf{r}_1) = \frac{1}{i\lambda} \int_{S_{\mathrm{A}}} U_{\mathrm{inc}}(\mathbf{r}_0) T(\mathbf{r}_0) \frac{\exp\left[ik\left(\mathbf{r}_{01}\right)\right]}{\mathrm{r}_{01}} \cos\left(\mathbf{r}_{01}, \mathbf{n}\right) dS, \tag{10}$$

where $U(\mathbf{r}_1)$ is the field in the observation plane, $U_{\mathrm{inc}}(\mathbf{r}_0)$ is the incident field in the diffraction plane, $T(\mathbf{r}_0)$ is the transmittance of the object. The coordinates of the observation plane are $\mathbf{r}_0 = (x_0, y_0, z = 0)$ and the coordinates of the observation plane are $\mathbf{r}_1 = (x_1, y_1, z = z_o)$, the vector $\mathbf{r}_{01}$ is the distance between two points in these planes, and $\mathbf{n}$ is normal to the surface of the object. Integration is performed over the surface of the element S_{A}.

We estimate the total transmission of the Gaussian and structured Airy THz illumination through a stratified sample with a Si wafer with refractive index n and a graphene layer with normalized conductivity σ/d, leading to refractive index n_{gr}. As the two-phase system is electrically connected, the depletion of graphene should lead to changes in the refractive index n of the Si wafer. For this reason, the depletion of graphene changes not only the optical refractive index n_{gr} of graphene but also the optical properties of the second phase. As the volume of graphene is small compared to the volume of the Si wafer, large changes in the refractive index n_{gr} will lead to relatively small changes in the refractive index n of the wafer. Transmission of the p and s polarized structured illumination through the stratified sample is depicted in Figure S2(a,b) for 3 different values of the refractive index of the substrate. We observe that, depending on the optical properties of the substrate, the transmission for the Airy beam can be larger than that of the Gaussian illumination.

Our main intention here is to explain the behavior that we observed in the experiment, so we investigate the ratio of two transmissions in Fig. S2(c). As the results indicate, for the s-polarization, the structured Airy illumination is mostly transmitted better than the Gaussian illumination as long as

the graphene is depleted. The optical properties of the wafer make this behavior less or more pronounced. Increasing conductivity diminishes this effect, and for refractive indices $n_{\mathrm{gr}} > 500$ the Gaussian illumination is transmitted better. We note that for $n_{\mathrm{gr}} > 200$ the number N of graphene layers becomes relevant. The results are also valid for the N layers if one keeps in mind that the optical path l_{opt} for one and N layers should be kept the same, i.e. $l_{opt} = n_N d_N = n_1 d_1$. The situation for p polarization is more dependent on the optical properties of the Si wafer. For the refractive index of Si-wafer $n = 3.50$ the Airy structured illuminations is transmitted better, whereas the decrease in the refractive index results in the better transmission of the Gaussian illumination through the imaging system. More results are given in the animated sequence presented as a Supplementary video S2.

These results can be summarized as a 2D plot of the transmission of the s-polarized Gaussian beam T_s^{Gauss}, see S3(a). As the p polarized Airy beam is transmitted in a way very similar to the s polarization, we present the difference caused by the structured illumination in Fig. S3(b) only for one case. Here we plot a ratio $T_s^{\mathrm{Airy}}/T_s^{\mathrm{Gauss}}$. In this way we observe that the difference in the behavior of the sample with 3 graphene layers can be caused either by inter-action of the graphene layer and the Si substrate, i. e. the changes in the graphene conductivity (meaning refractive index n_{gr}) or by the changes in the refractive index of the substrate n.

As results reveal, the substrate has a similar effect on s and p polarized THz radiation created by a ZP. Changes in the real part of the refractive index n cause an interferometric oscillation in the transmission for both polarizations. The difference between the s and p polarizations is not significant for the zone plate. However, for the structured bending THz illumination there is a distinct difference in comparison to the Gaussian signal. Thus, changes in the thickness of the wafer and in the optical properties of Si may also be the cause for the behavior we observed for the sample with 3 graphene layers.

The incident polarization in the experiment was y polarized and can be decomposed into s and p polarized as

$$\mathbf{e}_y = \mathbf{e}_s \cos\phi + \mathbf{e}_p \sin\phi, \tag{11}$$

where ϕ is the azimuthal angle of the polar coordinates in the xy plane. Therefore, the s component of the spatial spectra is $S(k_x, k_y)k_x/k$ and the p component is $S(k_x, k_y)k_y/k$. We were not fully certain how homogeneous the polarization state was; therefore, we have studied the s and p polarized components separately, and the transmission of the y polarized radiation is a weighted sum of the s and p polarized beams.

This set of simulation results hint at the fact that a) the set of signals for the ZP illumination sets some limits on the dielectric constant of the graphene and Si substrates in the stratified medium, and b) a comparison of the Gaussian signal with the Airy signal enables a more precise polarization-dependent determination of the dielectric constant or other parameters of the sample.

2 Raman mapping of graphene layers

Raman spectroscopy serves as a convenient tool for characterization of the quality of graphene. The main role is played here by two spectroscopic peaks – so-called G and 2D bands – in graphene Raman spectra that are located around 1580 cm^{-1} and 2690 cm^{-1}, respectively. The G band is associated with vibrations in the plane of carbon atoms bound to $SP2$. Its position, shape, and intensity are highly sensitive to the mechanical strain and doping. Mechanical stress may occur as a result of the interaction with the Si substrate on which graphene is transferred. The 2D band reflects a two phonon lattice vibrational process and it is always strong in graphene.

The Raman mapping was performed for the five samples studied that contained from 1 to 5 graphene layers. The mapping results of the G and 2D peaks are depicted on the left side of each section in Fig. S4(a-e). As one can see, the intensity of the G and 2D bands in the mapping of 3 layers of graphene is distinguished from others. Because the G band is strongly dependent of the mechanical strain, it can be assumed that during graphene transfer this sample was affected and thus experienced more strain between the layers in comparison to others, but not experiences carrier depletion. It also correlates with that seen in Fig.5(c) (in the main article): transmittance ratio in the sample consisting of 3 graphene layers increases in Airy lens experiment in comparison to the Gaussian one. It is related that the THz structured light is phase-sensitive, while the Gaussian THz illumination do not exhibits this feature. Therefore, the THz Airy imaging enables to reveal more precisely properties of single or several graphene layers placed on silicon substrates.

Moreover, existence of mechanical strain and doping can be exposed by correlation analysis of the frequency position of G and 2D bands in Raman spectra. The distribution of ω_{G} and ω_{2D} is shown on the right side of each segment in Fig. S4(a-e). The zero point indicates free graphene and is considered a constant (ω_{G}=1581.6±0.2 cm^{-1}; ω_{2D}=2676.9±0.7 cm^{-1}). Graphene interaction with Si substrate and each additional layer can cause a mechanical strain, while interaction with oxygen environment can affect graphene by hole doping.

In conclusion, the presence of mechanical strain and doping of graphene can be disclosed during inspection of the sample with structured THz light illumination.

A detailed study on the use of nonparaxial THz imaging and polarization-sensitive THz spectroscopy to characterize 2D materials is focus of a separate study to follow shortly.

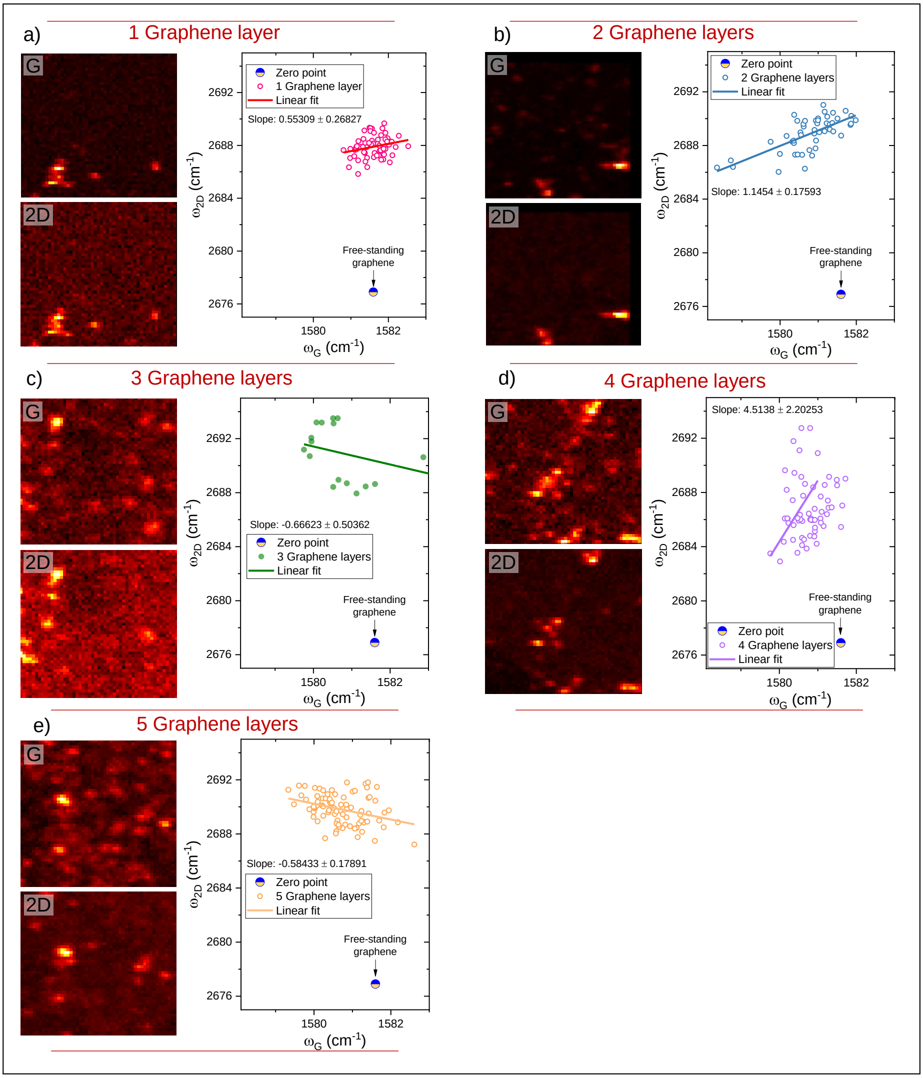

Fig. S4 Left panels: Results of the Raman mapping of G and 2D peaks for samples with different number of graphene layers. Note that for 1 and 2 graphene layers the map area displays low intensity intensity with only a few high intensity places. Sample containing 3 graphene layers expresses much higher intensity G and 2D peak mapping, meanwhile sample with 4 and 5 graphene layers intensity decreases, but still remaining higher than that of 1 and 2 graphene layers. **Right panels:** Distributions of ω_G and ω_{2D} peaks for each sample. Note that for the single graphene layer (a) distribution is relatively dense; for the sample containing 2 graphene layers (b) distribution of ω_G is wider indicating more strain. For 3 graphene layers sample (c), the slope changes its direction and frequency distribution of G and 2D peaks deviates stronger in comparison to other samples implying presence of a stronger mechanical strain. In case of 4 graphene layers (d), distribution of ω_{2D} gets wider meanwhile ω_G is dense, indicating, probably, presence of hole doping. The sample with 5 graphene layers (e), exhibits ω_G rather wide distribution implying mechanical strain between the layers.

References

[1] Lin, I.-T.: Optical Properties of Graphene from the THz to the Visible Spectral Region. PhD thesis, University of California, Los Angeles (2012)

[2] Yang, Y., Kolesov, G., Kocia, L., Heller, E.J.: Graphene terahertz absorption. arXiv preprint arXiv:1705.06267 (2017)

[3] Batrakov, K., Kuzhir, P., Maksimenko, S., Volynets, N., Voronovich, S., Paddubskaya, A., Valusis, G., Kaplas, T., Svirko, Y., Lambin, P.: Enhanced microwave-to-terahertz absorption in graphene. Applied Physics Letters **108**(12), 123101 (2016)

[4] Naumis, G.G., Barraza-Lopez, S., Oliva-Leyva, M., Terrones, H.: Electronic and optical properties of strained graphene and other strained 2d materials: a review. Reports on Progress in Physics **80**(9), 096501 (2017)

[5] Nashima, S., Morikawa, O., Takata, K., Hangyo, M.: Measurement of optical properties of highly doped silicon by terahertz time domain reflection spectroscopy. Applied physics letters **79**(24), 3923–3925 (2001)

[6] Pejcinovic, B.: Examination of silicon material properties using thz time-domain spectroscopy. In: 2014 37th International Convention on Information and Communication Technology, Electronics and Microelectronics (MIPRO), pp. 22–26 (2014)

[7] Hansen, W.N.: Electric fields produced by the propagation of plane coherent electromagnetic radiation in a stratified medium. JOSA **58**(3), 380–390 (1968)

[8] Born, M., Wolf, E.: Principles of optics: electromagnetic theory of propagation, interference and diffraction of light. Elsevier (2013)
