# Supplementary figures and images for: Terahertz structured light: nonparaxial Airy imaging using silicon diffractive optics
Source: Light Sci Appl. 2022 Nov 17;11:326. doi: 10.1038/s41377-022-01007-z (PMC9668966; doi:10.1038/s41377-022-01007-z)

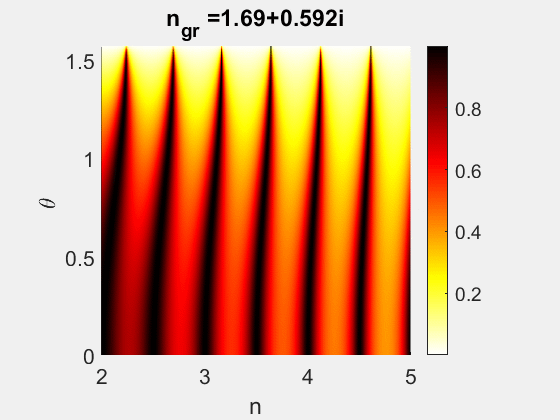

Supplement: Supplementary file 2 — Transmittivity for p-polarization [file 41377_2022_1007_MOESM2_ESM.gif]

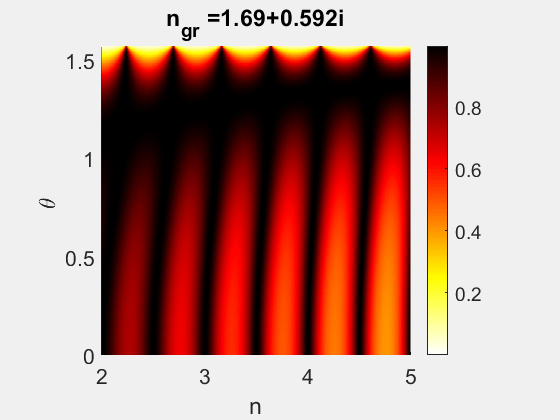

Supplement: Supplementary file 3 — Transmittivity for s-polarization [file 41377_2022_1007_MOESM3_ESM.gif]

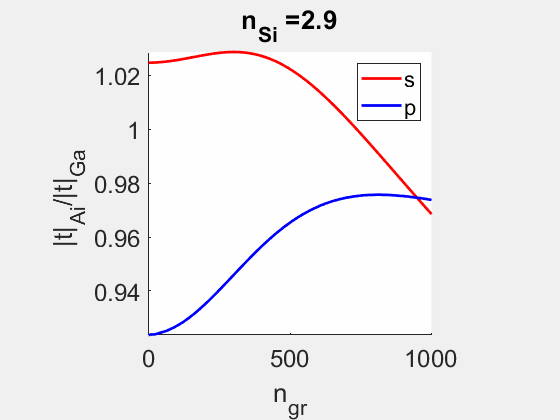

Supplement: Supplementary file 4 — Ratio of the transmission [file 41377_2022_1007_MOESM4_ESM.gif]
